# Robustness of replica symmetry breaking phenomenology in random laser

**DOI:** 10.1038/srep37113

**Published:** 2016-11-16

**Authors:** Federico Tommasi, Emilio Ignesti, Stefano Lepri, Stefano Cavalieri

**Affiliations:** 1 Dipartimento di Fisica e Astronomia, Università di Firenze, Via G. Sansone 1 I-50019 Sesto Fiorentino, Italy; 2 Istituto dei Sistemi Complessi, Consiglio Nazionale delle Ricerche, via Madonna del Piano 10, I-50019 Sesto Fiorentino, Italy

## Abstract

Random lasers are optical sources where light is amplified by stimulated emission along random paths through an amplifying scattering medium. Connections between their physics and the one of quenched disordered nonlinear systems, notably spin glasses, have been recently suggested. Here we report a first experimental study of correlations of spectral fluctuations intensity in a random laser medium where the scatterers displacement significantly changes among consecutive shots. Remarkably, our results reveal that the replica symmetry breaking (RSB) phenomenology is robust with respect to an averaging over different realizations of the disorder. Moreover, besides opening new intriguing questions about the understanding of such a phenomenon, this work aims to clarify the connection between the RSB with the onset of the Lévy regime, i.e. the fluctuations regime that is a peculiar feature of the random lasing under critical conditions. Our results suggest that the former occurs independently of the latter and then the RSB phenomenology is a generic feature linked to the random laser threshold.

The random laser^1^,^2^ is an optical source that has been extensively investigated during the last two decades. First theorized at the end of the 60s^3^, it has been experimentally realized starting from the mid 90s^4^,^5^,^6^,^7^,^8^,^9^. It consists of a gain medium with disordered structure capable of increasing the photons lifetime inside the active material, leading amplification by stimulated emission without the requirement of an optical cavity^10^. Such a system has a laser-like threshold fixed by the well known ‘gain larger than losses’-condition, that manifests as a sharp narrowing of the emission spectrum. The scattering mean free path *L*_*s*_ and the gain length *L*_*g*_ are the fundamental parameters characterizing, respectively, the degree of disorder and the non-linear effect of the amplification.

In random lasers, the interplay between non-linear feedback and the stochastic behaviour is crucial to determine the output emission. From a theoretical point of view, such a system can be described as a complex dynamics among a huge number of interacting modes^11^ in a way strongly dependent on the scattering and on the energy stored in the gain medium. A mode-coupling theory based on time-dynamics of frequency-locking mechanism has also been elaborated^12^,^13^. A complementary approach, describing photons as diffusing particles, has been also pursued. In this framework, it has been predicted that, under critical conditions, a possible exotic behaviour with fat-tailed distribution of fluctuation emission can exist. Such regime, known as *Lévy regime*^14^,^15^, is characterized by narrow random spikes that also appear different with the same starting conditions. This regime is at variance with the ones with smooth spectra (*Gaussian regimes*) characterizing the emission at low and at high pump energies. The coherent feedback due to randomly self-generated cavities among different displacements of scatterers and also Anderson localization has been proposed for strongly scattering media^16^,^17^. Another scenario has been theorized in weakly scattering systems, with extended modes that interact among them via the competition for the available gain^18^. In this picture, the origin of random peaks has a statistical meaning, with an incoherent non-resonant feedback that leads to a prevalence and to an exceptional growing of few long-lived modes among the others^19^,^20^. The statistical properties of these regimes have been studied^15^,^21^ as well as the presence of a crossovers among them^22^,^23^,^24^,^25^. A link between theory and experiment has been eventually found via the comparison between Monte Carlo simulations and the statistics of experimental emission spectra^26^,^27^. The *α*-exponent, obtained from the *α*-stable distribution fit of the peak intensity histogram, has been used as an indicator for characterizing the statistical regimes and also the threshold condition^28^.

An approach based on *spin glass theory*^29^, that describes out of equilibrium phenomena in disordered systems, has been used to investigate also the random laser dynamics^30^,^31^. In this context, experimental evidence of *replica symmetry breaking* (RSB) in a random laser has been recently reported^32^, revealing and underlying non-trivial modes organization and correlation. RSB consists in a change of the statistical distribution of the *overlap parameter* (Parisi Overlap), that describes how different states of a system interact and emerge. In presence of RSB, system replicas, i.e. identical realizations under the same experimental conditions, yield different values of the observable parameters. Within this picture, this system is composed by many equivalent degenerate states, with a glassy behaviour^33^ that manifests as a thermodynamic phase where the symmetry among different replicas is broken. The overlap parameter assumes the role of an order parameter that measures the correlation (anti-correlation) between intensity fluctuations, as the different amount of disorder and non-linear effect are introduced in the random medium. Hence, the spin-glass theory, besides the other proposed theoretical models, can be successfully applied to describe the random laser dynamics^34^.

In its first experimental realisation^32^, the glassy-behaviour above threshold was observed in a strongly scattering solid-state sample, where the experimentally accessible quantities are the fluctuations of the emission spectrum. It is important to stress that up to now the solid state structure of the active material was considered as a fundamental requirement for describing of the random laser as a *quenched disordered* system^35^. A second work reported the observation of RSB in a random laser system using strongly scattering solid media (*L*_*s*_ of the order of tens of μm)^36^. Such a work also investigated the intriguing possibility to find a link between the RSB and the onset of the Lévy regime. Moreover, also a decaying trend of the spin-glass behaviour at high pumping energies was reported. Recently, also an observation of RSB in specially designed TiO_2_ particle-based dye-colloidal random laser was provided^37^. RSB in a fluctuating regime at lasing threshold of a liquid state dye lasers *without disorder* has been even reported^38^.

Here we report an experimental measurement of the intensity fluctuations overlap, a parameter that has been introduced to demonstrate the symmetry breaking between different replicas in quenched disorder systems. The system under study is a liquid random laser, where the condition of identical realization of the disorder is *specifically not fulfilled*. Remarkably, our results show that the RSB phenomenology emerges even in a medium as arranged. Moreover, we report the appearing of RSB both in the case of Lévy or Gaussian regime. The RSB transition appears as linked to the random laser threshold, independently on whether the Lévy regime is present or not.

## Experimental Results

The experimental set-up (see the Methods section) consists of a pumping system provided by a pulsed laser, a liquid sample for the random laser emission and a synchronized system to collect a large number of spectra. The system as arranged is able to associate at each random laser emission spectrum the corresponding pump pulse energy. The liquid samples consist of a solution of dye in ethanol with ZnO nanoparticles as light-scattering sources. Changing their concentration it is possible to tune the scattering mean free path *L*_*s*_. The values of *L*_*s*_ have been measured as described in the Sample Preparation subsection. We considered three different samples having *L*_*s*_ equal to 19.0 mm, 7.44 mm and 900 μm, respectively. The time interval between two consecutive excitation events by the pump pulse is 200 ms and each set of measurements is ~10^3^ s-long.

The experiments on RSB in random lasers reported up to now have been carried out focusing on the assumption that disorder is quenched i.e. not changing in time. In practice, this requires that^34^: (i) the interaction strengths among modes (i.e. the random coupling constants) changes on a time scale much longer than the typical random laser lifetime and (ii) they remain unchanged in successive series of measurements on the same sample. Both requirements are obviously met in solid media. However, for our liquid samples only condition (i) is satisfied (the relevant timescale being 10 ns). To demonstrate this, we performed a measurement of the speckles pattern fluctuations induced by a coherent light propagation through our active medium with moving particles. A He-Ne beam was shone on a cuvette filled with ZnO nanoparticles in ethanol and the speckles pattern was observed on a screen placed after the scattering medium. We acquired a 5 s long video, i.e. a time corresponding to 25 consecutive excitation events. Indeed, the speckles pattern changed position on time scale shorter than that of two consecutive spectral acquisitions, meaning that the arrangement of scatterers was different. A dedicated discussion of this issue is reported in section Methods - Scatterers Motion Estimation. In other words, we are performing a measurement over an ensemble of different disorder configurations and each of them can be considered as quenched on the time scale of each measurement.

For each measurement session a pumping energy is selected and, in order to ensure identical conditions for the excitation, only spectra produced by a pump energy that lies within a prescribed range (deviations of 2% from the mean value) are selected. Moreover, the spectral window considered is a 70 nm long interval centred on the emission peak, in order to remove the noise due to the low-intensity wings of the spectrum. In turn, each interval is binned in *N*_*b*_ values resulting form the finite resolution of the spectrometer (0.22 nm), i.e. including the modes with similar wavelength in the *b*-th bin. These selected spectra for each measurement session are the *N*_*s*_ system “replicas” used for the calculation of the distribution *P(q*) of the *N*_*s*_(*N*_*s*_ − 1)/2-values of the overlap parameter *q*_*αβ*_^32^,^34^:


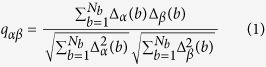


where *α* and *β* are the indexes that label the different replicas and the Δ_*i*_(*b*) are the fluctuation of the intensity *I*_*i*_(*b*) of the *i*-th replica in the *b*-th bin around the mean value 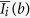 in the same bin of all spectra 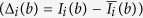. In this work, the value of *N*_*s*_ used for the calculation for each pumping energy ranges between 2000 and 4000.

**Figure 1:**
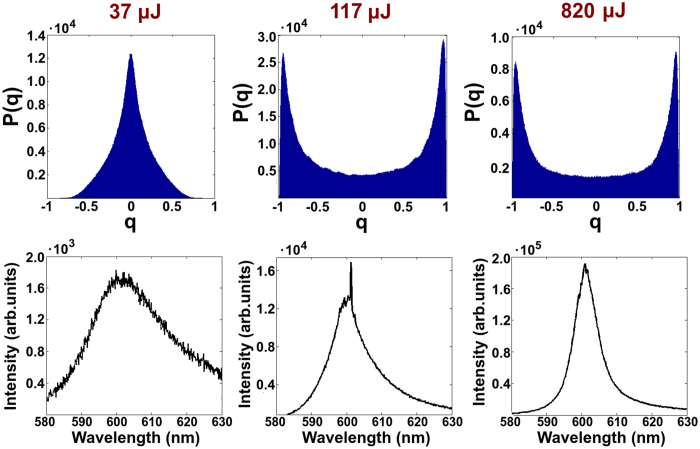
Distribution functions (above) of the overlap for the sample with *L*_*s*_ = 7.44 mm. Distributions of *q* for three different pumping energies that correspond to three different statistical regimes: 37 μJ (*First Gaussian*), 117 μJ (*Lévy*) and 820 μJ (*Second Gaussian*). Below, typical emission spectra are reported.

**Figure 2:**
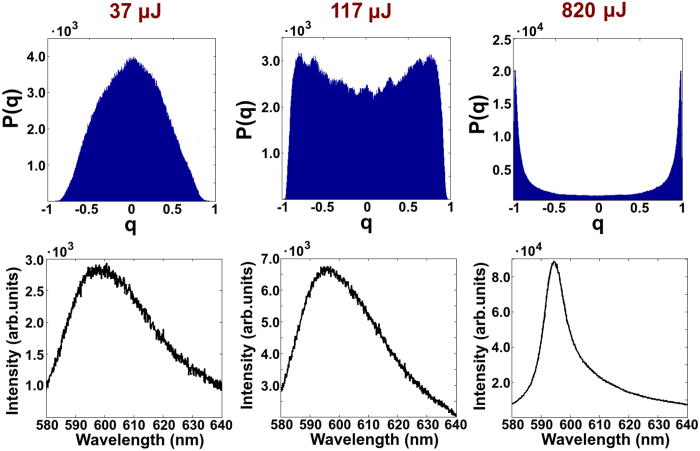
Distribution functions (above) of the overlap for the sample with *L*_*s*_ = 900 μm. Distributions of *q* for three different pumping energies that, since the *Lévy regime* is not present for this sample, in any case correspond to a *Gaussian regime*: 37 μJ, 117 μJ and 820 μJ. Below, typical emission spectra are reported.

In the upper panels of Figs 1 and 2 the overlap distributions *P(q*) for different energies and for the samples with *L*_*s*_ = 7.44 mm and 900 μm respectively are shown. The trend of *P(q*) for the two cases is qualitatively the same: at low energy the spectra are determined by spontaneous emission and *P(q*) has a maximum around *q* = 0: the modes do not interact. As the energy increases, *P(q*) broadens, covering the interval [−1, 1] until it displays two humps close to the interval extrema: the modes strongly interact. Thus, RSB phenomenology upon increasing the pumping energy is also experimentally demonstrated for liquid scattering samples where the realization of the disorder changes.

In the lower panels of Figs 1 and 2 the typical emission spectra are reported. In the first case, i.e. the sample with a weakly scattering (Fig. 1), for the intermediate range of pumping energies the emission spectrum shows a fluctuations regime with random spikes. On the contrary, in the second one (Fig. 2) the spectrum undergoes narrowing without passing through a Lèvy fluctuations regime. As the characterization of *α* reported below will show, the three levels of pump energy correspond to three different statistical regimes (First Gaussian, Lévy, Second Gaussian) in the first case and two in the second one (First and Second Gaussian). Our previous works^21^,^26^,^27^ have described in detail that these statistical regimes depend on the pumping energy and the scattering strength of the samples; in particular, a critical role is played by the ratio between *L*_*s*_ and the linear dimension *w* of the active zone of the medium. In the case of 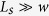, the weak modes coupling given by a moderate competition for the available gain, leads to a broadening of the energies interval involved by the Lévy regime. Upon increasing *L*_*s*_, a stronger coupling is established, leading to a narrowing of the Lévy regime until its complete inhibition. When present, such a regime emerges near threshold, i.e. once the stimulated emission becomes a significant process compared to the spontaneous one. In such a case rare long-lived modes can acquire a lot of energy and appear as energetic random spikes on top of a broad emission spectrum. Thus, it is evident that near threshold the *α*-stable fit leads to lowest vales of *α*, because at higher energies a strong coupling emerges due the larger number of modes^26^.

In order to clarify the connection of the RSB with the random laser threshold and the Lévy regime, an extensive experimental characterization has been carried out. Hence, a statistical analysis of the same sets of spectra used for the calculation of *P(q*) has been performed. The ensemble of values obtained from the *α*-stable fit^39^,^40^,^41^ are made by selection for each spectrum of the 5 bins that are around the peak value of the averaged spectrum. All data and not only the tail-values are used for the fitting. A Lévy regime can be considered when the value of *α* falls below 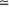
1.8, given the large but finite number of considered spectra.

**Figure 3:**
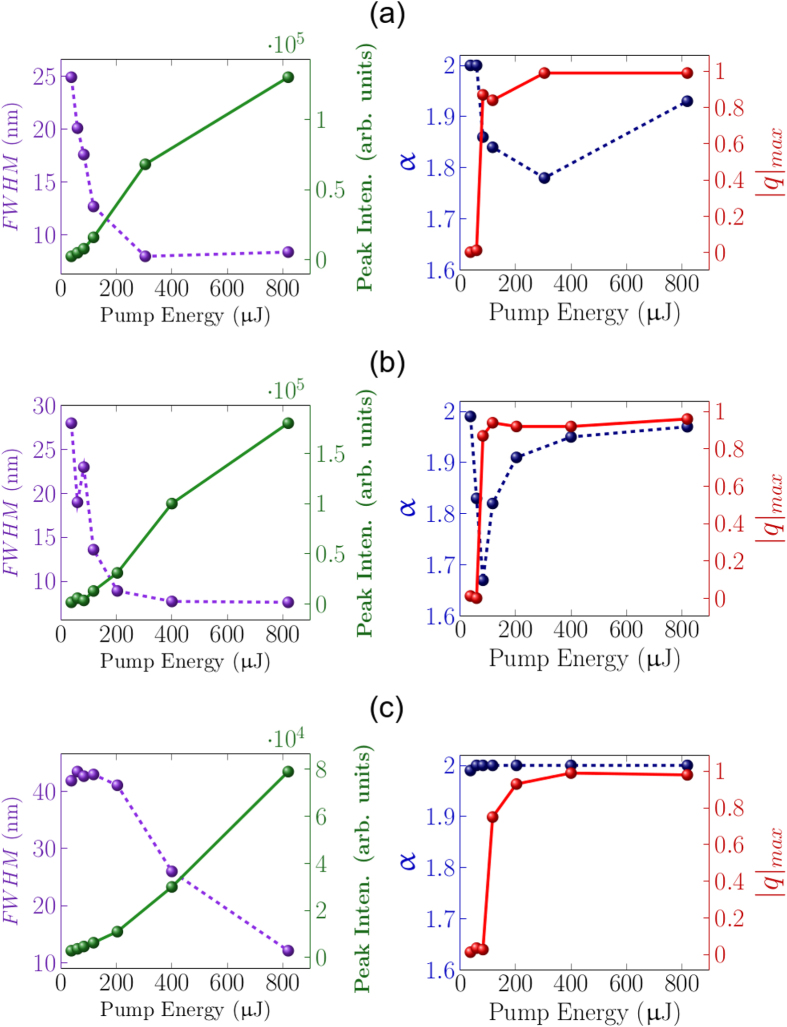
Characterization of random laser emission. Characterizations as a function of pumping energy of the emission from the three samples: *L*_*s*_ = 19.0 mm (**a**), 7.44 mm (**b**) and 900 μm (**c**). In the left column the FWHM (purple, left y-axis) and the peak intensity (green, right axis) are reported. In the right one the *α*-index (blue, left y-axis) and the |*q*|_*max*_ are shown. The threshold energy can be detected in correspondence of the narrowing of the spectrum or in the change of the slope of the peak intensity plot. An abrupt change of |*q*|_*max*_ can be found at threshold, in a way independently on the presence of the Lévy regime 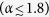.

In the left column of Fig. 3 for the three samples (*L*_*s*_ = 19.0 mm (1), 7.44 mm (2) and 900 μm (3)) the FWHM and the peak intensity of the averaged spectrum, i.e. the mean over all spectra of each set, are reported for increasing energies. Unlike a conventional laser, the random laser threshold is not as well defined and alternative approach has been investigated^42^. However, a reasonable estimate can be given from both the spectral narrowing and the slope changing of the peak intensity as the pumping energy increases. Indeed, such an effect is due to the prevalence of the stimulated emission over the spontaneous one, giving the ‘gain larger than losses’-condition.

In the right column of Fig. 3 the |*q*|_*max*_ and *α* as the function of energy for the same samples are shown. The former is the value that correspond to the peak of the distribution of *P*(|*q*|) and establishes the value for the overlap for each set of spectra. As the energy increases, an abrupt change of *P*(|*q*|) reveals the RSB condition and appears, for all samples, around the threshold detected by the characterization reported in the left column of Fig. 3. The figures also show that the RSB appears in a way independent on the presence of the Lévy regime.

To conclude this section, we note that in the work reporting the first evidence of this phenomenology in random lasers^32^ no RSB was detected in liquid samples. In such an experiment, the pump intensity was much lower (less than four orders of magnitude) than the one considered here, yielding a much weaker nonlinear coupling among modes. We surmise that this may be the cause of the observed differences.

## Discussion

The evidence of RSB in random lasers has opened an intriguing new opportunity to understand their physics and to connect it to the statistical mechanics of disordered nonlinear systems.

In the present work we reported an important extension, where disorder is different shot-to-shot: the dynamics of the random laser has indeed a time scale much shorter than scatterers motion in a single shot, but the arrangement of scatterers evolves appreciably during the whole measurement. In other terms, the characteristic time-scales of the experiment are such that many realizations of disorder are probed within a measurement under the same conditions. The phenomenology of RSB transition, namely the onset of multiple peaks in *P(q*), is clearly observed. Admittedly, this observation lacks a theoretical justification. At a first glance, the experimental protocol entails an averaging over disorder realizations, reminiscent of what is done in spin-glass simulations^43^. However, both the averaging procedure and the overlap (1) are somehow different from what used there. Also, the possibility of multiple peaks in the *P(q*), measured as done here, appears to be ruled out by the theory (see the discussion in ref. 34). A further theoretical effort is needed to find an explanation.

Another point we focused on is the connection between the Lévy regime and the triggering of the RSB transition, as recently reported^36^. Hence, we studied a sample where a Lévy regime does not occur, because the comparison between the active zone dimension and *L*_*s*_ in this case does not allow it, as previous works has reported^26^,^27^. Although the lack of strong fluctuations in such a sample as the energy increase (α ~ 2), the threshold is detectable in the energy interval within which the RSB sets in. Therefore, the RSB and the Lévy regime onset, that emerges above the same critical conditions fixed by the *L*_*s*_ and *L*_*g*_, may not have a clear causal relation between them. A deeper study of this issue, both experimental and theoretical, is thus required.

## Methods

### Sample Preparation

The samples consist in 1 mM solution of Rhodamine B dye in ethanol with different concentrations of ZnO nanoparticles with a mean average diameter of 35 nm. In order to estimate *L*_*s*_, the extinction coefficient *μ*_*e*_ has been measured as a function of the scatterers concentrations (0.17 ÷ 2.6% of mass fraction), in a turbid medium composed by ZnO nanoparticles in ethanol. The attenuation of the ballistic beam intensity *I*_0_ in a medium of thickness *z* is given by the Lambert-Beer law:


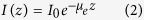


The used experimental set-up^44^ consists in a chopped 10-mW He-Ne laser that provides the beam that is sent to the turbid medium contained in a cuvette with a depth of 10 mm. The output light passes through a diaphragm, to suppress the contribute of the scattered light, and eventually detected by a photodiode. The signal is measured by a lock-in amplifier connected to the chopper. The wavelength of the He-Ne laser (632.8 nm) is near the maximum of the emission of the Rodhamine B in ethanol. Neglecting the contribute of absorption at 632.8 nm of the ethanol^45^ (~3 · 10^−4^ mm^−1^) and the ZnO, 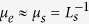. The reported values of *L*_*s*_ of the three samples investigated in this work have a relative uncertainty of 2%.

A numerical calculation by Mie theory, assuming monodisperse spherical non-absorbing particles, leads to values close to the experimental ones by fixing a radius of 21.3 nm (*L*_*s*_@632.8 *nm* = 19.1, 7.48 and 0.908 mm). The calculation has been performed by a code based on the BHMIE subroutine of the book of Boheren and Huffman^46^. The manufacturer of the ZnO particles reports polydispersion with an average particles radius of 17.5 nm with an upper limit of 75 nm. With the same radius, the values for the transport mean free path *L*_*T*_@632.8 *nm* become 19.4, 7.60 and 0.922 mm. The small radius of the particles causes a scattering strengthening at the pump wavelength (*L*_*s*_@532 *nm* = 9.54, 3.73 and 0.453 mm and *L*_*T*_@532 *nm* = 9.75, 3.82, 0.463 mm). Hence for the third sample the scattering of the pump photons should cause a broadening of the active zone of the sample. Then, in the sample used in this work the diffusion regime condition, i.e. *L*_*s*_ much larger than the wavelength, is well fulfilled.

### Experimental Set-up

**Figure 4:**
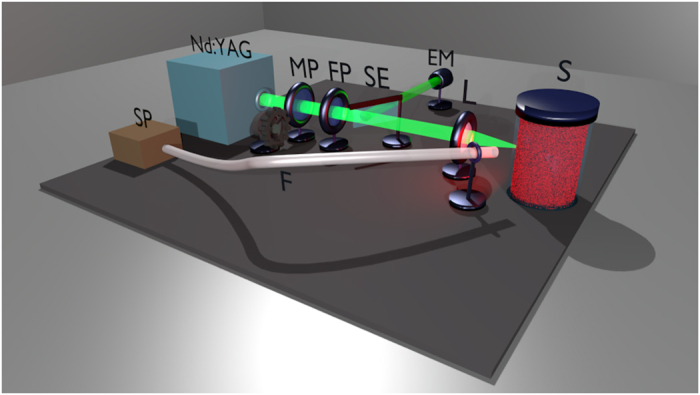
Scheme of the experimental set-up. Nd:YAG: pumping beam source; MP: movable polarizer; FP: fixed polarizer; SE: semi-transparent plate; EM: energy meter; L: lens; F: optical fibre; SP: spectrometer; S: sample

Figure 4 shows a schematic diagram of the experimental set-up. The pump beam, provided by a frequency doubled (532 nm) Q-switched Nd:YAG at a repetition rate of 5 Hz, is tuned in energy by a pair of polarizers, one moveable (MP) and controlled by a stepper motor and one fixed (FP), and focalised to the cuvette (S) filled with the liquid sample. Each pulse is ~5 ns-long. The focal spot diameter on the sample is ~100 μm. A reflection from a semi-transparent plate (SE) is sent to an energy meter (EM) connected to a PC to measure the energy of each pump pulse. The random laser emission is collected by an optical fibre, whose head is placed at a distance of ~10 cm from the cuvette and with an angle of ~15° with respect the pump beam direction. The spectrometer (SP) is an Ocean Optics USB 2000+ with a resolution of 0.22 nm.

The generation of the pump pulses, the measurement of their energy and the acquisition of each random laser emission spectrum are synchronized by the trigger signal provided by the control unity of the Nd:YAG, controlled by a PC and automatized. Then, the system as arranged is able to store a large number of spectra with the corresponding pumping energy. Before each measurement session, MP is set to provide the desired pump energy. Then, the shot-to-shot energy variations in the same session are only due to the intensity fluctuations of the Nd:YAG.

### Scatterers Motion Estimation

We think that is important to stress here the different conditions of motion of the scatterers in our experiment compared to the previously reported ones. The peculiar time scales of the experiment are the random laser lifetime, in our case few ns, and the total time of acquisition of the spectra (tens of minutes). The motion of the scatterers is frozen in the RL lifetime, in such a way that the condition of quenched disorder is fulfilled in this time scale.

Completely different is the characteristics of the sample in the total time of acquisition; in particular the speckle movements (see video in Supplementary Material) show that in the sample the scatterers move at least on the spatial scale of the wavelength during the total time of acquisition or even from shot to shot. Indeed, as it usually occurs in systems where modes are due to interaction of light along different optical paths, the change in the speckles pattern between two consecutive shots indicates that the condition of identical realizations of the disorder should not be fulfilled from the point of view of the propagating radiation.

**Figure 5:**
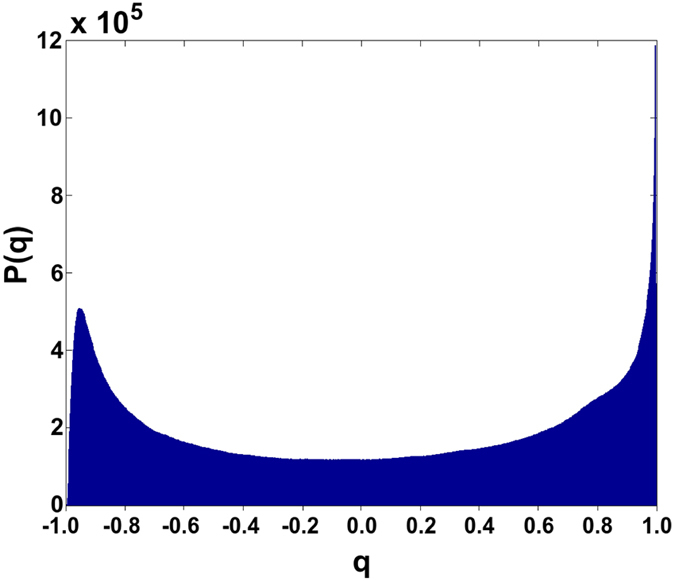
Supplementary ‘longtime’-measurement. Parisi overlap distribution upon two consecutive sets of measurement that covered about four hours of the sample lifetime. The pump energy (≈800 μJ) is far above threshold and the total number of spectra is 2 · 10^4^. Despite the motion of the scatterers, RSB is evident.

However, one could also reasonably suppose that the relevant spatial scale for the motion of the scatterers is of the order of *L*_*s*_^37^. In the results shown in Fig. 3 the Brownian motion travelling length (*L*_*B*_), during the total time of acquisition, is comparable to the values of *L*_*s*_ (from ~1 ÷ 20 mm) in the different samples. In addition, to stress further this condition we performed a dedicated ‘long-time’-measure (~4 hours); in such a case *L*_*B*_ (1.5 mm) is larger than *L*_*s*_ (900 μm) and the distribution of *P(q*)) is still not peaked on q = 0 (see Fig. 5). In other words, also if the sample is not “frozen” on the scale of *L*_*s*_ the RSB phenomenology is evident.

## Additional Information

**How to cite this article**: Tommasi, F. *et al.* Robustness of replica symmetry breaking phenomenology in random laser. *Sci. Rep.* **6**, 37113; doi: 10.1038/srep37113 (2016).

**Publisher's note**: Springer Nature remains neutral with regard to jurisdictional claims in published maps and institutional affiliations.

## Supplementary Material

Supplementary Information

Supplementary Video 1
