# Supplementary material for: Robustness of replica symmetry breaking phenomenology in random laser
Source: Sci Rep. 2016 Nov 16;6:37113. doi: 10.1038/srep37113 (PMC5111062; doi:10.1038/srep37113)
Supplement: Supplementary Information [file srep37113-s1.doc]

**Robustness of replica symmetry breaking phenomenology in random laser**

**F. Tommasi, E. Ignesti, S. Lepri, S. Cavalieri**

The Supplementary file contains Supplementary Videos 1 and its Legend.

**Supplementary Video 1:**

**Fluctuations of speckles pattern due to movement of particles**

Slow motion video of the fluctuations of the speckles pattern in a screen placed after a cuvette, through which a He-Ne laser beam propagates . The cuvette is filled with ethanol and dispersed ZnO nanoparticles. The ballistic beam is attenuated in the centre of the screen. The original video is 5 seconds long, i.e. the time corresponding to 25 consecutive spectral acquisitions of different replicas. The frame rate is decreased from 30.015 to 15.015 frames/s.
